# Case Occurring in Stanton Military General Hospital—Surg. John A. Lidell, U. S. V. in Charge, Gun-shot Wound of Head; Punctured Fracture of Right Parietal Bone—Recovery

**Published:** 1863-10

**Authors:** Wm. H. Gail

**Affiliations:** Medical Cadet U. S. A.


ART. II.—*Case occurring in Stanton Military General Hospital—Surg.* JOHN A. LIDELL, U. S. V. *in charge. Gun-shot Wound of Head; Punctured Fracture of right Parietal Bone—Recovery. Reported by* WM. H. GAIL, *Medical Cadet U. S. A.*

Corporal Ezra Scarborough, Co. G, 15th New Jersey Volunteers, admitted to Stanton Hospital May 8th, 1863, aged 34 years, and of robust constitution; was wounded in battle at Fredericksburg May 3d, 1863, by a musket shot; fell down insensible on the receipt of the injury, and remained unconscious for some time—how long he does not know. Examination disclosed a gun-shot wound of the scalp, near the vertex, and a little to the right of the median line, complicated with depressed fracture of the skull in the same locality. The depressed portion of bone was about one inch long by one-half of an inch in width, and was located in the right parietal bone, on the vertex near the sagittal suture. Amount of depression about one-fourth of an inch. The left lower extremity and the left arm were both paralyzed at the time of his admission. Could flex the fingers of the left hand a little, but in no other respect could move any part of the extremities; paralysis was of motion, and not of sensation; the muscles of the face were not paralyzed, and he protruded his tongue well; he exhibited some confusion of intellect, some tardiness in answering questions, but was not delirious. Complained of some headache, a dull pain shooting from the wound towards the right eye; pupils contracted and symmetrical, but sluggish to stimulus of light; no unnatural heat of head; pulse about seventy per minute, soft and full; skin soft, moist, and natural in temperature; bowels confined; has never vomited; wound was already suppurating.

*Treatment*—cut off the hair close; water dressing to wound; ice bag to head, a saline purgative, and a low diet. Under this treatment the case progressed favorably; had slight headache almost continually; there was a constant tendency to constipation, so that it was frequently necessary to administer purgatives.

By the 15th of May, the paralysis had diminished so much that he could flex the left elbow. At this time his bowels did not move without the aid of medicines; pupils still contracted and sluggish; pulse ranges from sixty-five to seventy per minute.

May 25th.—Has recovered good use of the left arm, but it is not yet as strong as the other; the depressed bone is partially elevated; pupils not contracted, but sluggish; pulse continues to range from sixty-five to seventy.

June 7th.—A fragment of the fractured bone has become detached, and was removed this morning; it proved to be a piece of the internal table of the skull, nearly one inch in length by one-half an inch in width; the dura-mater became visible at the bottom of the wound, together with the pulsations of the brain; pupils natural in size, and contracting readily under the stimulus of light; tongue clean; secretions normal; wound suppurating properly, looks healthy, and has already contracted some; ice bag discontinued; other treatment same as before.

June 12th.—Pulse has risen in frequency from fifty-five to eighty; paralysis of left leg diminishing in a marked degree.

June 24th.—Paralysis continues to diminish; has complained of headache at irregular intervals, especially if the bowels were confined.

July 8th.—Wound of head gradually closing up; pulsations of brain no longer visible; patient begins to leave his bed.

July 17th.—Two small pieces of bone detached, and lying in bottom of wound, removed to-day; patient doing well in every respect.

August 1st.—Patient doing well; wound of scalp nearly closed; walks with the aid of a cane.

August 17th.—Patient left the hospital on furlough.

These cases are submitted without comment. The great diversity of opinion existing, and that has existed among the most eminent surgeons the world has ever produced, regarding the operation of trephining, induces me to present them without remark, and let every one draw his own conclusions.

The cases are interesting; the history complete and impartial, and may serve to illustrate the truth of Liston's aphorism, that "no injury of the head is too slight to be despised, or too severe to be despaired of."

